# Everybody's Page

**Published:** 1891-07-11

**Authors:** 


### EVERYBODY'S PAGE.

IF the progress of the Sunday Society is slow, its members are able to congratulate themselves on the fact that considerable modifications have, during recent years, been made in the method of regarding the day in this country. The Sabbatarian spirit is still strong in our midst as evidenced by the shrieking and fuss made at the prospect of the German Emperor visiting the Naval Exhibition; but the power of these holy and pious people who, as Professor Romanes puts it, seem to prefer the British Sunday of the slums, with its deadening vacuity, its gluttony, and its debauchery, to the innocent and refining influence of art and literature, is on the wane. The public are beginning to awake to the knowledge that the object of the Sunday Society is to enhance and not to lessen the value and importance of the Sunday.

PROFESSIONAL begging, especially in large and wealthy cities, is no doubt a very paying business. It has much to recommend it. It requires no capital but impudence and plausibility. Many of these gentlemen and ladies are probably richer than those from whom they solicit alms. Mendicity has proved such a flourishing trade in Paris, that serious efforts are at length being made to abolish it. The plan which it appears is to be adopted is, however, more simple than it is likely to prove efficacious. It is proposed to open an office in each arrondisement of the City, to which all people of good character who are unable to earn their living can apply for work or assistance, which, after due investigation, will be granted. There is nothing novel in this, and it is hardly likely to stop coppers flowing into the hats of the professional beggar from the pockets of those who prefer to give their penny rather than take the trouble of inquiring into the genuineness of the appeal. So long as human nature remains as it is there is little prospect of stamping out the mendicant.

COMPETITION in the medical profession appears to be as great in the United States as in this country, and to bring in its trail many undesirable effects. If, as Dr. Edwin A. Lewis, of New York, considers, people are in the habit of employing and changing their physician much as they do a grocer or butcher, it is most improbable that a doctor who knows he is the third one employed by a family in as many months will take the personal interest in any given case that he would if he were the regular family attendant. Dr. Lewis is not fond of books of the nature of "Every Man His Own Doctor," which lead the public to believe that it is the happy possessor of important medical knowledge, when it is in reality worse off than if it pretended to none at all. By means of such works the general public is not only over-educated but badly educated on medical and surgical matters.

A WRITER in the *New York Medical Record* holds up the modern cigarette-smoker to opprobrium. The inhalation of tobacco-smoke, which has become such a prevalent practice during recent years, is, he considers, a new morbid habit and a peril which confronts society. The inhalation of the smoke introduces into the system quickly and delightfully a narcotic poison, and awakens in the smoker a sensation as pleasant as that produced by opium. "The seriousness of the cigarette to inhalation lies not alone in the fact that it involves a steady absorption of the poison, but in the utter hopelessness of the habit, and the entire inability of the indulger to give it up. Once a cigarette inhaler, always one. In this respect it resembles with painful similarity the opium habit. One may stop the use of pipe or cigar, or the use of tea, beer, or whiskey, but the morphine and tobacco inhalation habits, is well-established, are practically incurable." So says the American; but, as usual, he exaggerates.

